# A Hybrid Generalized Hidden Markov Model-Based Condition Monitoring Approach for Rolling Bearings

**DOI:** 10.3390/s17051143

**Published:** 2017-05-18

**Authors:** Jie Liu, Youmin Hu, Bo Wu, Yan Wang, Fengyun Xie

**Affiliations:** 1 School of Mechanical Science and Engineering, Huazhong University of Science and Technology, Wuhan 430074, China; eijuil@hust.edu.cn (J.L.); youmhwh@hust.edu.cn (Y.H.); 2 Woodruff School of Mechanical Engineering, Georgia Institute of Technology, Atlanta, GA 30332, USA; yan.wang@me.gatech.edu; 3 School of Mechatronics and Vehicle Engineering, East China Jiaotong University, Nanchang 330013, China; xiefyun@163.com

**Keywords:** condition monitoring and fault diagnostics, state recognition and classification, feature extraction and reduction, signal decomposition, generalized interval

## Abstract

The operating condition of rolling bearings affects productivity and quality in the rotating machine process. Developing an effective rolling bearing condition monitoring approach is critical to accurately identify the operating condition. In this paper, a hybrid generalized hidden Markov model-based condition monitoring approach for rolling bearings is proposed, where interval valued features are used to efficiently recognize and classify machine states in the machine process. In the proposed method, vibration signals are decomposed into multiple modes with variational mode decomposition (VMD). Parameters of the VMD, in the form of generalized intervals, provide a concise representation for aleatory and epistemic uncertainty and improve the robustness of identification. The multi-scale permutation entropy method is applied to extract state features from the decomposed signals in different operating conditions. Traditional principal component analysis is adopted to reduce feature size and computational cost. With the extracted features’ information, the generalized hidden Markov model, based on generalized interval probability, is used to recognize and classify the fault types and fault severity levels. Finally, the experiment results show that the proposed method is effective at recognizing and classifying the fault types and fault severity levels of rolling bearings. This monitoring method is also efficient enough to quantify the two uncertainty components.

## 1. Introduction

Rolling bearings are important and fragile parts in machinery. As the connection between the rotor and the support, the safety and stability of rolling bearings are the key to ensure the normal operations of machines. Thus, it is very important to diagnosis the rolling bearings fault at its incipient stage in order to prevent long-term breakdowns or in some cases possibly catastrophic failures.

In recent years, bearing health monitoring research has attracted considerable attention [1,2,3,4,5,6,7,8,9]. The vibration analysis method has been widely applied for diagnosing the rolling bearing fault due to its intrinsic merits of revealing bearing failure. Different signal processing methods, such as traditional spectral decomposition methods, such as wavelet transform [10], empirical mode decomposition (EMD) [11], ensemble empirical mode decomposition [12], etc., in both the time and frequency domains have also been employed to analyze the collected non-stationary vibration signals and to extract relevant and sensitive features. However, the major challenges in these methods include the selection of basis functions, handling mode mixing and end effects, and the removal of background noise. The variational mode decomposition (VMD) is an adaptive and entirely non-recursive signal decomposition method recently proposed by Dragomiretskiy and Zosso [13]. VMD can extract the principal mode of the signal and the respective center frequencies. It shows superior performance in signal decomposition and feature extraction.

The outcome of VMD is affected by the typical selection of the number of components *K* and the weight or balancing parameter α. These two parameters need to be set before analyzing the vibration signal. Some research has been investigated to deal with this problem. For example, Wang et al. find that a small value of the α parameter will be used for the purpose of detecting impacts [14]. Zhu et al. present an adaptive VMD method with artificial fish swarm algorithm-based parameter optimization for detecting the localized faults of the rolling bearing [15]. Yi et al. propose a novel method of fault feature extraction based on the combination of VMD and the particle swarm optimization algorithm [16]. Zhang et al. predefine the number of the modes *K*, the balancing parameter of the data-fidelity constraint α using the correlation and the energy ratio function [17]. In these methods, parameters with precise values are calculated. These approaches are robust enough, especially when the working condition is changed.

It has been argued that there is no need to obtain precise parameters. Zhang et al. find that one or several additional modes would greatly contain noise when small α and large *K* or large α and small *K* are chosen. Additionally, significant parts of the spectrum are shared by two or more different modes, and their center frequencies overlap when small α and small *K* or large α and large *K* are used [18]. Thus, to some extent, the confounding effects of these two parameters are not precisely known. A more robust approach is not predetermining precise values of the parameters in advance and allowing for perturbation in applications. Liu et al. find that the number of frequencies of interest helps to determine the parameter *K* based on the power spectrum under the machine state [19]. The selection of balancing parameter α is usually set in a searching range. Therefore, epistemic uncertainty as a result of the lack of knowledge is significant and cannot be ignored. In this paper, the uncertainty associated with the parameter is considered, and the balancing parameter α is set in the form of intervals $\left[\underline{\alpha },\bar{\alpha }\right]$. It helps to quantify the epistemic uncertainty.

Naturally, after extracting feature vectors, the multi-fault classifier is needed to automatically conduct the fault diagnosis, which is common in practical systems [20]. For example, the hidden Markov model (HMM) has the capability of statistical learning and classification and has been widely applied in some condition monitoring and diagnosis applications. However, it cannot differentiate two types of uncertainties. Aleatory uncertainty is from inherent randomness and also known as random error, variability or irreducible uncertainty, whereas epistemic uncertainty is due to lack of knowledge and known as systematic error, incertitude or reducible uncertainty [21]. One approach to capture these two uncertainty components is to characterize uncertainty with imprecise probability so that we can improve the robustness of decision making. Recently, Wang proposed a new generalized interval probability to simplify the calculation of imprecise probability [22]. Compared to the classical interval, it has better algebraic and semantic properties and provides an intuitive framework for applications of interval probability. Based on the generalized interval probability, a generalized hidden Markov model (GHMM) was proposed [23]. The GHMM was developed to enhance reasoning where aleatory uncertainty is represented as probability; intervals are used to capture epistemic uncertainty. It improves the reliability of recognition with more information provided by the interval probability values than the HMM.

In this paper, a hybrid generalized hidden Markov model-based condition monitoring (GHMM-CM) approach is proposed. The major contributions of this paper include:

1. Rolling bearing feature extraction from noise-contaminated sensor signals based on VMD and GHMM is proposed firstly to improve the reliability of recognition with more information provided by the interval probability values;
2. Selection of balancing parameter α is set in the form of the generalized intervals $\left[\underline{\alpha },\bar{\alpha }\right]$ to quantify the epistemic uncertainty;
3. System errors that are inherent during data collection for the learning and feature recognition stage are incorporated.

The paper is organized as follows: Section 2 provides the overview of relevant work. The architecture of the hybrid GHMM-CM process is illustrated in Section 3. Experimental results in the rotating machine process are analyzed to verify the performance of the proposed method in Section 4. Finally, this paper is concluded with a summary of the proposed method.

## 2. Background

### 2.1. Variational Mode Decomposition

VMD is a newly-developed methodology for adaptive and quasi-orthogonal signal decomposition [13]. In the VMD framework, the signal is decomposed into *K* discrete number of sub-signals, and each component is considered compact around a corresponding center frequency. The process of the VMD can be considered as a constrained optimization problem, formulated as:

(1) $$\begin{array}{c} \min_{\left\{u_{k}\right\},\left\{\omega_{k}\right\}}\left\{\sum_{k=1}^{K}∥\partial_{t}\left[\left(\delta \left(t\right)+\frac{j}{\pi t}\right)*u_{k}\left(t\right)e^{-j\omega_{k}t}\right]{∥}_{2}^{2}\right\},s.t.\sum_{k=1}^{K}u_{k}\left(t\right)=f\left(t\right), \end{array}$$

where each mode $u_{k}$ is almost compact around a matching center frequency $\omega_{k}$, and its bandwidth is assessed by means of $H^{1}$ Gaussian smoothness. *K* is the number of decomposed sub-signals. $u_{k}$ is the decomposed sub-signal. f(t) is the original vibration signal.

Equation (1) can be solved via the augmented Lagrangian method:

(2) $$\begin{array}{cc} L & (\left\{u_{k}\right\},\left\{\omega_{k}\right\},\lambda )=\alpha \sum_{k=1}^{K}∥\partial_{t}\left[\left(\delta \left(t\right)+\frac{j}{\pi t}\right)*e^{-j\omega_{k}t}\right]{∥}_{2}^{2}\\ & +∥f\left(t\right)-\sum_{k=1}^{K}u_{k}\left(t\right){∥}_{2}^{2}+\langle \lambda \left(t\right),f\left(t\right)-\sum_{k=1}^{K}u_{k}\left(t\right)\rangle . \end{array}$$

Alternating direct multipliers are typically adopted to solve Equation (2). The estimated modes $u_{k}$ and the corresponding updated center frequency $\omega_{k}$ in the frequency domain can be written as:

(3) $$\begin{array}{c} {\hat{u}}_{k}^{n+1}\left(\omega \right)=\frac{\hat{f}\left(\omega \right)-\sum_{i\neq k}{\hat{u}}_{i}\left(\omega \right)+\frac{\hat{\lambda }\left(\omega \right)}{2}}{1+2\alpha {\left(\omega -\omega_{k}\right)}^{2}}, \end{array}$$

(4) $$\begin{array}{c} \omega_{k}^{n+1}=\frac{\int_{0}^{\infty }\omega {\left|{\hat{u}}_{k}\left(\omega \right)\right|}^{2}d\omega }{\int_{0}^{\infty }{\left|{\hat{u}}_{k}\left(\omega \right)\right|}^{2}d\omega }. \end{array}$$

where α is the balancing parameter of the data-fidelity constraint. More specifically, the process and more details of the VMD algorithm can be found in [13].

### 2.2. Multi-Scale Permutation Entropy

After VMD decomposition, a major current focus is how to extract the fault information from the obtained main components. Many studies have been conducted to investigate the feature extraction methods. For example, permutation entropy (PE) measures the complexity through comparing the neighboring values; it is simple, immune to noise and suitable for online monitoring of the mechanical system [24]. Based on the PE method, multiscale permutation entropy (MPE) was used to estimate the complexity of the time series in different scales [25]. Furthermore, the advantages of MPE have been validated, such as stability and robustness. In addition, Wu used MPE to diagnose the rolling bearing fault and verified that the MPE has better performance compared with PE [26]. Therefore, MPE is taken as a feature extractor to extract the fault information from the vibration signals in this paper. Given an N-length time series, x(t),t=1,2,⋯,N, the m-dimensional vector $X_{i}^{m}$ at time *i* is defined as:

(5) $$\begin{array}{c} X_{i}^{m}=\left[x\left(i\right),x\left(i+\tau \right),\cdots ,x\left(i+\left(m-1\right)\tau \right)\right],i=1,2,\cdots ,N-\left(m-1\right)\tau , \end{array}$$

where *m* is the embedding dimension and τ is time delay. If the vector $X_{i}^{m}$ satisfies that:

(6) $$x\left(t+k_{0}\tau \right)⩽x\left(t+k_{1}\tau \right)⩽\cdots ⩽x\left(t+k_{m-1}\tau \right),$$

where $0⩽k_{i}⩽m-1$ and $k_{i}\neq k_{j}$, it has a permutation $\pi_{k_{0},k_{1},\cdots ,k_{m-1}}$.

Later, the relative frequency of each permutation π can be defined by:

(7) $$\begin{array}{c} p\left(\pi \right)=\frac{Number\{X_{i}^{m}\}}{N-(m-1)\tau }. \end{array}$$

After that, the permutation entropy with mdimension can be defined by:

(8) $$\begin{array}{c} H_{PE}\left(m\right)=-\sum p\left(\pi \right)\ln\left(p\left(\pi \right)\right). \end{array}$$

When all possible permutations appear with the same probability, the maximal $H_{PE}\left(m\right)$ with a value of ln(m!) is obtained. Then, the normalized permutation entropy (NPE) can be expressed by:

(9) $$\begin{array}{c} H_{NPE}\left(m\right)=\frac{H_{PE}\left(m\right)}{\ln(m!)}, \end{array}$$

where $0⩽H_{NPE}\left(m\right)⩽1$. From the above process, PE can be utilized to detect the dynamic change of the time series.

A coarse-grained procedure is proposed to obtain multiple scale time series from the original time series [27]. Then, the entropy at each scale is calculated to analyze the complicated signal. Given a time series, x(t),t=1,2,⋯,N, a consecutive coarse-grained time series $y^{\left(s\right)}$ with a time scale *s* is constructed by:

(10) $$\begin{array}{c} y_{j}^{\left(s\right)}=\frac{1}{s}\sum_{i=(j-1)s+1}^{js}x_{j},1⩽j⩽\frac{N}{s}, \end{array}$$

where the time series is divided into non-overlapping windows of length *s*.

To overcome this shortcoming, based on the concepts of multi-scale and PE, the MPE was proposed to calculate entropy over multiple scales [25]. In MPE analysis, the entropy of the coarse-grained time series at each scale is calculated by the NPE algorithm.

### 2.3. Principal Component Analysis

After extracting the fault features using MPE, the obtained features are fed into the multi-fault classifier to accomplish the fault diagnosis. However, the feature vectors obtained from vibration signals using MPE are high-dimensional with information redundancy, which will reduce the diagnosis accuracy. An effective approach to select m most important scale factors to construct the fault feature vectors is necessary. Thus, a lightweight dimensionality reduction approach is needed to overcome this challenge. In this work, a dimensionality reduction approach based on the traditional principal component analysis (PCA) technique is used to process signals decomposed by the VMD method. The approach aims to choose the most important features that exhibit high divisibility and contain the most important fault information from the obtained generalized features of the MPE.

Dimensionality reduction is one of the preprocessing steps in practical condition monitoring and fault diagnosis applications. The lower dimension space represents the direction of the maximum variance of the given features, which makes it suitable for some fast condition monitoring applications. The PCA technique, as one of the most used unsupervised dimensionality reduction techniques, finds relationships between observations and transforms high-dimension features into a lower dimension space [28].

Using the PCA technique, dimensionality reduction removes redundant information and interference noise existing in the feature set. Thus, the most sensitive and important information is extracted from the raw features.

### 2.4. Generalized Hidden Markov Model

With both the aleatory and epistemic uncertainty considered, a generalized interval probability-based GHMM structure was proposed [22,29]. The aleatory uncertainty is represented as probability; the interval captures the epistemic uncertainty; and all probability parameters of HMM are replaced by the generalized interval probabilities. The boldface symbols have generalized interval values. The GHMM is characterized as follows. The values of hidden states are still in the form of $S=\{S_{1},S_{2},\cdots ,S_{N}\}$, where *N* is the total number of possible hidden states. The hidden state variable at time *t* is $q_{t}$. The *M* possible distinct observation symbols per state are $V=\{v_{1},v_{2},\cdots ,v_{M}\}$. The observation sequence is in the form of $O=(o_{1},o_{2},\cdots ,o_{T})$, where $o_{t}$ is the observation value at time *t*. Note that the observations have the values in the form of generalized intervals. Events of the observation sequence $O=(o_{1},o_{2},\cdots ,o_{T})$ can be directly observed. In contrast, hidden states sequence $Q=(q_{1},q_{2},\cdots ,q_{T})$ cannot be observed directly, but can be inferred by the observation sequence. Let $q_{t}\in pro\left[{\underline{q}}_{t},{\bar{q}}_{t}\right]$ and $o_{t}\in pro\left[{\underline{o}}_{t},{\bar{o}}_{t}\right]$ be real-valued random variables that are included in the respective interval-valued random sets $\left[{\underline{q}}_{t},{\bar{q}}_{t}\right]$ and $\left[{\underline{o}}_{t},{\bar{o}}_{t}\right]$.

$A={\left(a_{ij}\right)}_{N\times N}$ is the state transition probability matrix, where $a_{ij}:=\left[{\underline{a}}_{ij},{\bar{a}}_{ij}\right]$ is the generalized interval probability; ${\underline{a}}_{ij}$, ${\bar{a}}_{ij}$ are the lower and upper transition probability from state $S_{i}$ at time *t* to state $S_{j}$ at time t+1. Specifically:

(11) $$\begin{array}{cc} {\underline{a}}_{ij} & =\underline{p}\left(q_{t+1}=S_{j}|q_{t}=S_{i}\right))⩾0,\sum_{j=1}^{N}{\underline{a}}_{ij}=1,\left(1⩽i,j⩽N\right),\\ {\bar{a}}_{ij} & =\bar{p}\left(q_{t+1}=S_{j}|q_{t}=S_{i}\right)⩾0,\sum_{j=1}^{N}{\bar{a}}_{ij}=1,\left(1⩽i,j⩽N\right). \end{array}$$

$B={\left(b_{j}\left(k\right)\right)}_{N\times M}$ is the observation probability matrix with $b_{j}\left(k\right):=\left[{\underline{b}}_{j}\left(k\right),{\bar{b}}_{j}\left(k\right)\right]$ in state $S_{i}$, where $b_{j}\left(k\right)$ is the generalized interval probability, ${\underline{b}}_{j}\left(k\right)$, ${\bar{b}}_{j}\left(k\right)$ are the lower and upper observations probability in state $S_{j}$ at time *t*. Specifically:

(12) $$\begin{array}{cc} {\underline{b}}_{j}\left(k\right) & =\underline{p}\left(o_{t}=v_{k}|q_{t}=S_{j}\right)⩾0,\sum_{j=1}^{M}{\underline{b}}_{j}\left(k\right)=1,\left(1⩽j⩽N,1⩽k⩽M\right),\\ {\bar{b}}_{j}\left(k\right) & =\bar{p}\left(o_{t}=v_{k}|q_{t}=S_{j}\right)⩾0,\sum_{j=1}^{M}{\bar{b}}_{j}\left(k\right)=1,\left(1⩽j⩽N,1⩽k⩽M\right). \end{array}$$

$\pi ={\left(\pi_{i}\right)}_{1\times N}$ is the initial state probability distribution, where $\pi_{i}:=\left[{\underline{\pi }}_{i},{\bar{\pi }}_{i}\right]$ is the generalized interval probability; ${\underline{\pi }}_{i}$, ${\bar{\pi }}_{i}$ are the lower and upper probability in state $S_{i}$ at t=1. Specifically:

(13) $$\begin{array}{cc} {\underline{\pi }}_{i} & =\underline{p}\left(q_{1}=S_{i}\right)⩾0,\sum_{i=1}^{N}{\underline{\pi }}_{i}=1,\left(1⩽i⩽N\right),\\ {\bar{\pi }}_{i} & =\bar{p}\left(q_{1}=S_{i}\right)⩾0,\sum_{i=1}^{N}{\bar{\pi }}_{i}=1,\left(1⩽i⩽N\right). \end{array}$$

The parameters *N* and *M* should be predefined. The proposed GHMM is denoted as λ={A,B,ß} with $\lambda :=[\underline{\lambda },\bar{\lambda }]$, where $\underline{\lambda }=\left\{\underline{A},\underline{B},\underline{\pi }\right\}$ and $\bar{\lambda }=\left\{\bar{A},\bar{B},\bar{\pi }\right\}$. The GHMM can describe different stochastic processes by the definition of A,B and ß under different probability distributions.

The GHMM can also solve three kinds of problems, i.e., evaluation, decoding and learning. The GHMM learning adopts the maximum log-likelihood to update model parameters. The relationship of the observation sequence with the optimal state sequence is referred to as the decoding process. Combining with the generalized interval probability [22,29], the evaluation, decoding and learning processes in the GHMM can be achieved. The algorithms include the generalized forward-backward algorithm, generalized Viterbi algorithm and generalized learning algorithm. More details about these algorithms can be referred to the previous works [23,29,30,31].

## 3. Methodology

**Figure 1:**
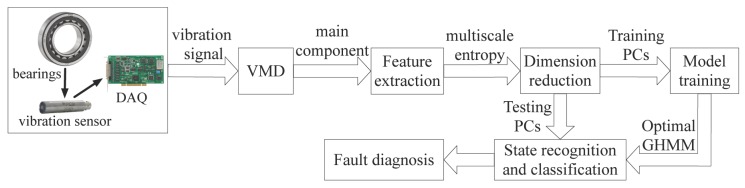
Basic flow of the proposed hybrid generalized hidden Markov model-based condition monitoring (GHMM-CM) method.

Figure 1 shows the basic flow chart of the hybrid GHMM-CM method. First, the VMD method is used to decompose the vibration signals and obtain a set of sub-signals. Here, the balancing parameter is predefined and set in the form of generalized intervals $\left[\underline{\alpha },\bar{\alpha }\right]$. Epistemic uncertainty caused by the lack of knowledge is considered. Later, the MPE is calculated to realize generalized interval-based features extraction of the principal decomposed signal. After that, the PCA technique is applied to reducing the dimensionality of features and computational cost. Next, the initial GHMM of the training datasets is established, and the optimal GHMM of each state is obtained by the generalized learning algorithm. Then, the extracted features of the testing datasets are used as inputs of the optimal GHMM. Based on the generalized interval probability, the optimal GHMM enhances the reasoning of aleatory uncertainty and epistemic uncertainty. More information is provided to improve the reliability of the states’ recognition.

Finally, fault types and fault severity levels of rolling bearings are recognized and classified by the proposed hybrid GHMM-CM method, and the fault diagnosis of the rotation process is realized.

## 4. Experimental Verification

### 4.1. Experimental Setup

**Figure 2:**
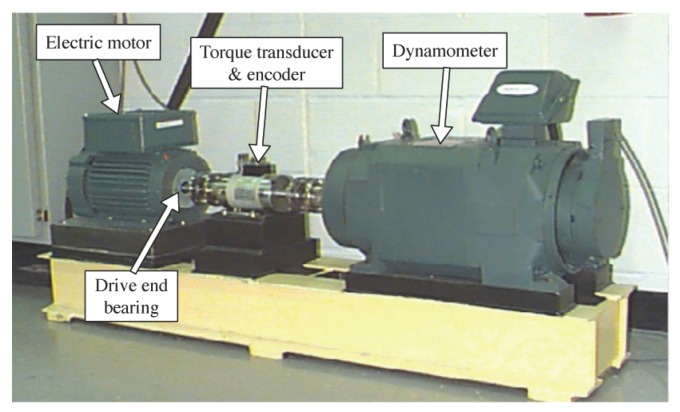
Experimental setup of the rolling bearings for Case Western Reserve University (CWRU) [32].

To validate the effectiveness of the proposed method, public experimental data from the bearing data center of Case Western Reserve University (CWRU) were analyzed [32]. The photograph of the experimental setup is shown in Figure 2, in which the 6205-2RS JEM (SKF, Gothenburg, Sweden) deep groove ball bearing is used. The vibration signals of the rolling bearing were collected under three fault types including inner race defect (IRD), ball defect (BD) and outer race defect (ORD). The fault bearings were using the electro-discharge machining with fault diameters of 7, 14, 21 and 28 mils to simulate different fault severities. The associated rotating speed of driving motor was set to 1730 rpm, 1750 rpm, 1772 rpm and 1797 rpm, respectively. Accelerometers were placed at the 12 o’clock position of the driving motor end to measure the vibration signals under different working conditions. The sampling rate was set to 12 kHz. The relevant data settings of experiments are illustrated in Table 1.

In the following experiments, recognition and classification for different fault types are performed. The fault diameter used in the experiments is 21 mil, and the motor speed is 1750 rpm with 2 Hpload. Vibration signals are divided into non-overlapping segments with the length *N* = 2400. Each machine state has 50 samples, in which 25 samples will be chosen as the training dataset using the Kennard and Stone algorithm [33,34]. The remaining 25 samples are used to test the constructed system model. All algorithms were processed by MATLAB 9.1.0 (2016b, MathWorks Inc., Natick, MA, USA) in a laptop with an Intel Core i5 CPU and 8G RAM (Apple Inc., Cupertino, CA, USA).

### 4.2. Signal Analysis

For the bearing with fixed outer race, there are some fundamental frequencies, which are defined as follows [35].

(14) $$\begin{array}{c} \mathrm{Inner}\;\mathrm{race}\;\mathrm{defect}\;\mathrm{frequency}:f_{id}=\frac{Nf_{r}}{2}\left(1+\frac{d}{D}\cos\alpha \right), \end{array}$$

(15) $$\begin{array}{c} \mathrm{Ball}\;\mathrm{defect}\;\mathrm{frequency}:f_{bd}=f_{r}\frac{D}{d}\left(1-\frac{d^{2}}{D^{2}}\cos^{2}\alpha \right), \end{array}$$

(16) $$\begin{array}{c} \mathrm{Outer}\;\mathrm{race}\;\mathrm{defect}\;\mathrm{frequency}:f_{od}=\frac{Nf_{r}}{2}\left(1-\frac{d}{D}\cos\alpha \right), \end{array}$$

where $f_{r}$ is the shaft rotation frequency, *d* is the ball diameter, *D* is the pitch diameter, *N* is the number of rolling elements and α is the contact angle.

**Figure 3:**
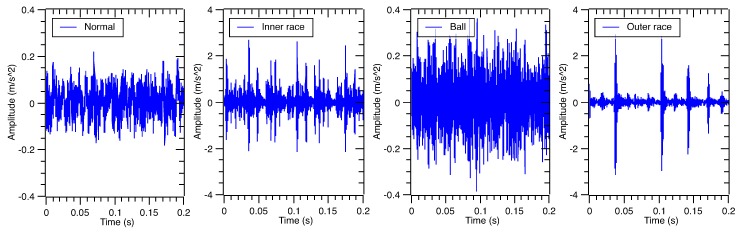
The waveforms of the rolling bearing vibration signal under four different conditions.

Based on the geometrical parameters shown in Table 2, corresponding bearing defect frequencies under the rotating speed 1750 rpm are shown in Table 3. Vibration signals of accelerated sensors under four fault categories in the time domain are illustrated in Figure 3, respectively.

**Figure 4:**
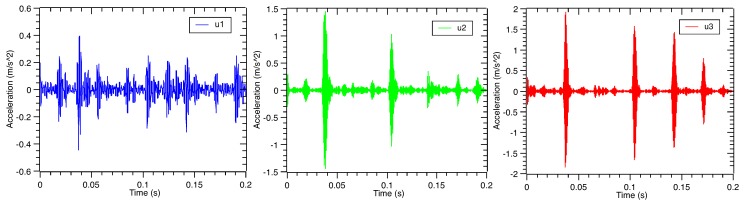
The time domain waveforms of rolling bearing vibration signal under the outer race defect after the variational mode decomposition (VMD) process.

**Figure 5:**
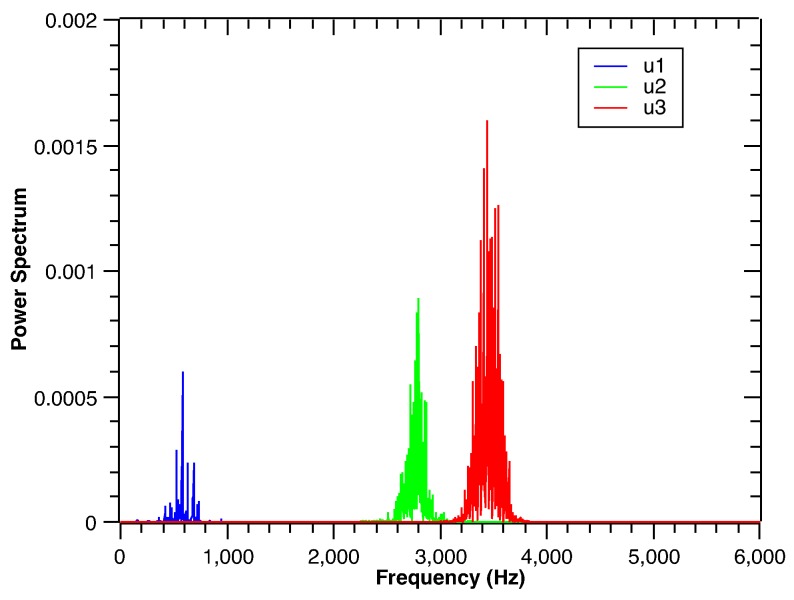
The frequency domain waveforms of rolling bearing vibration signal under the outer race defect after the VMD process.

Due to the nonlinear and non-stationary characteristics of vibration, the VMD method is used to decompose the vibration signal under four bearing defects. The vibration signal under the outer race defect in Figure 3 is taken as an example. The decomposition results are shown in Figure 4. At the same time, corresponding fast Fourier transform of the decomposed sub-signals is illustrated in Figure 5. Typically, the spectrum energy of the outer race defect, inner race defect and rolling element defect vibration signal is concentrated in the natural frequency. Here, the defect vibration signal is dominated by a high frequency oscillation waveform, which carries the information about the impulse response of the structure. It can be found that the third decomposed sub-signal corresponds to the high frequency oscillation waveform. However, traditional fast Fourier transform (FFT) cannot describe the defect features. Using the Hilbert transform, the envelope analysis method can effectively extract different location defect features from the vibration signal of the rolling bearing [36]. The Hilbert transform of the vibration signal is defined by:

(17) $$\begin{array}{c} \hat{x}\left(t\right)=\frac{1}{\pi }\int_{+\infty }^{+\infty }\frac{x(\tau )}{t-\tau }d\tau . \end{array}$$

The complex analytic signal z(t) can be obtained by:

(18) $$\begin{array}{c} z\left(t\right)=x\left(t\right)+i\hat{x}\left(t\right), \end{array}$$

where x(t) is the origin vibration signal and $\hat{x}\left(t\right)$ is the Hilbert transform of the origin vibration signal, $i^{2}=-1$.

**Figure 6:**
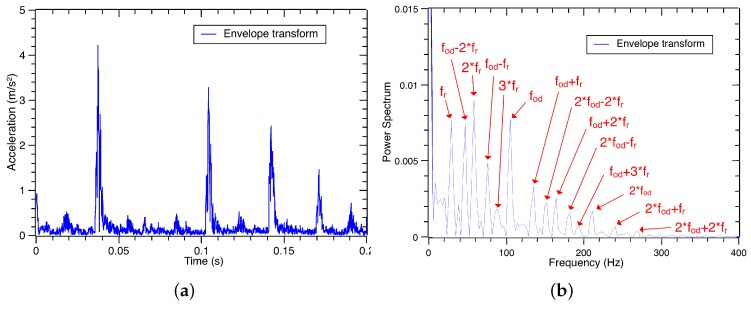
The envelope waveform and spectrum of the raw vibration signal under the outer race defect.

**Figure 7:**
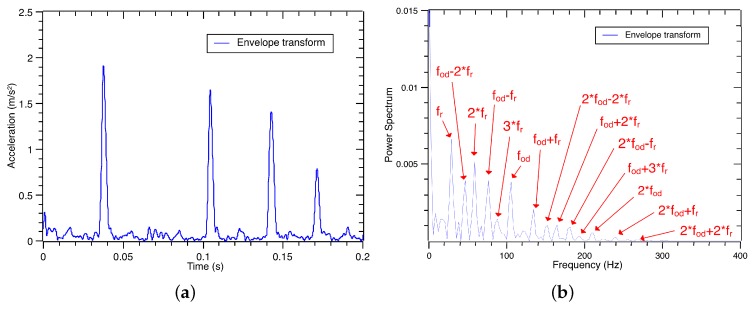
The envelope waveform and spectrum of the third decomposed sub-signal under the outer race defect.

Using Equations (17) and (18), the envelope waveform of the raw vibration signal is obtained, and its frequency spectrum can be calculated by the FFT. The envelope waveform and its spectrum are shown in Figure 6a,b. Similarly, the envelope transform of the third decomposed sub-signal is shown in Figure 7a,b. Based on the envelope waveform output in Figure 6a,b, noise and interference are filtered out in the third decomposed sub-signal by the VMD process. Through the energy in the decomposed signal is reduced, the interested characteristic frequencies of the outer race defect are successfully retained in the envelope spectrum as shown in Figure 7b. The signal-to-noise ratio is improved by the VMD process. Thus, the third decomposed sub-signal can be used to recognize and classify the vibration signal of the defect bearings.

### 4.3. Signal Decomposition

Typical selection of the modes number *K* and the balancing parameter α affects the outcome of the VMD. Before, the signal decomposition, initialization and input parameters of the VMD need to be predefined. Thus, they are key parameters of VMD [14,18,37]. Some parameter optimization methods have been investigated in [15,16,38,39]. In practical applications, these methods increase the computational cost especially under different operation conditions. In this paper, these two parameters are defined by prior knowledge. On the one hand, the number of modes *K* can be manually set based on the frequency distribution of the vibration signals. Parameter *K* cannot be less than the number of frequencies of interest. Based on the analysis in Section 4.2, suitable parameter *K* is set to three in this paper.

On the other hand, the selection of balancing parameter α is usually set in a searching range. In the VMD, the balancing parameter α controls the data preservation [17]. It determines noise levels of the decomposed component in the frequency domain and also affects the bandwidth of the matching center frequency. Thus, the exact balancing parameter α causes one common drawback. Here, the balancing parameter α is set in the form of the generalized intervals $\left[\underline{\alpha },\bar{\alpha }\right]$. It helps to quantify the epistemic uncertainty in the proposed hybrid GHMM-CM method. For example, for an exact balancing parameter α with the value of 1600, the generalized intervals are set to [1200, 2000] by general error ±25% in this paper. The Lagrangian multiplier was effectively shut off. The parameter ω was set to one, which means that center frequencies of all of the modes were initialized in the uniform distribution. No DC part was imposed.

### 4.4. Reduced Features Extraction

After the VMD process, the principal decomposed sub-signal contains sufficient information of the fault in rolling bearings. The time series with multiple scale structures are complicated. Before using the MPE, some setting of the parameters should be defined in advance, such as time series length *N*, embedding dimension *m*, time scale factor *s* and time delay τ. The embedding dimension *m* determines the accessible states. Christoph et al. indicated that the reasonable range of the embedding dimension is 3⩽m⩽7 [24]. Obviously, when *m* is too large, the calculation will be very resource intensive and time consuming. In contrast, when *m* is too small, it cannot work [40]. Thus, trade-offs have to be assessed on practical applications. In this paper, the embedding dimension *m* can be chosen as four. Moreover, the time series length *N* should satisfy the condition N⩾5m! [40]. Therefore, *N* is set to 2400, which is the data length of a sample. In the end, time scale factor *s* and time delay τ are set to 20 and one.

Using the MPE technique, the entropy of the coarse-grained time series at each scale is calculated. Therefore, features with fault information are obtained from the main decomposed sub-signal.

In this paper, a PCA-based dimensionality reduction approach is adopted to process these high-dimensional high-volume features. The PCA projects the entropy-based features to a lower dimensional space and ensures the major variance captured. At the same time, the problem of how many PCs need to be obtained should be determined. Typically, the extracted PCs represent the main characteristics of the original decomposed sub-signals. However, reducing the features too much makes it difficult to build the system model. Based on prior knowledge, the extracted PCs should generally capture at least 90.0% of the total variance.

**Figure 8:**
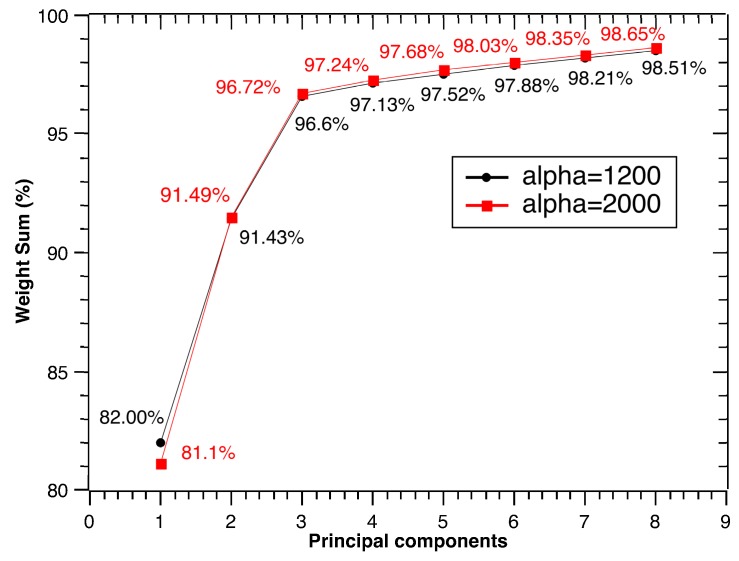
Robustness of the PCA space under parameter interval $\alpha =[\underline{\alpha },\bar{\alpha }]$.

The robustness of the PCA space is shown in Figure 8. When the upper balancing parameter $\bar{\alpha }$ is 2000, the total of the variance values using one PC to eight PCs is displayed in the red rectangle line. At the same time, when the lower balancing parameter $\underline{\alpha }$ is 1200, the total variance values are plotted in the black ellipse line. It can be found that the first three PCs capture 96.7% of the total variance under the generalized balancing parameter α. That is, the first three generalized PCs represent the main characteristics of the original decomposed sub-signals. Fault types of defect bearings will be recognized and classified based on the calculated generalized PCs in the next subsection. At the same time, the effects of different major PCs will also be discussed in detail later.

### 4.5. Fault Diagnosis

Considering the aleatory uncertainty and epistemic uncertainty simultaneously, an initial model λ is assumed, where number of states is four. Using the MPE, reduced generalized features are regarded as the observations of the GHMM. Here, the Lloyd algorithm [41] is used to encode the observation vector, and the observation sequence $O=(o_{1},o_{2},\cdots ,o_{8})$. The initial models of four fault types are trained and updated by the GHMM learning algorithm. Four GHMMs with respect to the four fault types are trained, and four optimal models are established. The observation sequences of the testing datasets are obtained, and then, they are substituted in the optimal GHMM. Four values of $\log p\left(O|{\tilde{\lambda }}_{i}\right)\left(i=1,2,3,4\right)$, which correspond to four optimal GHMMs respectively, for the testing datasets are calculated. The maximum log-likelihoods of the fault types in testing datasets under different optimal GHMMs are compared. In this paper, the maxi-min criterion (pessimistic criterion) of the interval comparison is used to improve the reliability of the estimation results [42].

In this criterion, the minimal result for each interval will be chosen firstly. Then, the maximal one within these minimal results is selected. For example, four samples from four fault types of defect bearings are tested, and the recognition results are shown in Table 4. Based on the maxi-min criterion, the minimal values of each interval in sample No. 1, such as −0.0842, −Inf, −582.69, −Inf, are selected. Then, the maximal one −0.0842 with these selected minimal values is chosen. Thus, the interval [−0.0842, −0.0412] is the maximum log-likelihood, which means the normal condition is recognized in Sample No. 1. Similarly, Test Sample Nos. 2, 3, 4 can be also identified by the proposed method.

Without loss of generality, the Kennard and Stone algorithm [33,34] was used to choose training and testing datasets from raw datasets with different fault types. In the experiments, there are four fault types of defect bearings in the rotation process. Thus, the outputs of the GHMM respond to the normal condition (NC), inner race defect condition (IRDC), ball defect condition (BDC) and outer race defect condition (ORDC), respectively. At the same time, the issue of sensitivity variance using different numbers of major generalized PCs is investigated. Different numbers of major generalized PCs are chosen as inputs of the GHMM.

The simulation results are shown in Table 5. On the one hand, the proposed method can recognize and classify all fault types of training datasets using 3, 4, 5, 6, 7 and 8 PCs. On the other hand, the accuracy rate of testing datasets is no less than 98% using the proposed methods with 4, 5, 6, 7 and 8 PCs. However, limited PCs cause information loss. There is also a trade-off in choosing the number of generalized PCs. Based on the practical applications, the proposed method not only maintains the accuracy rate, but also reduces the computation cost. Based on the simulation results above, the performance of the proposed hybrid GHMM-CM method using reduced features is demonstrated.

### 4.6. Discussion

#### 4.6.1. Improvement in Signal Decomposition

In the proposed hybrid GHMM-CM method, the improvements in signal decomposition help to recognize and classify the fault types of defect bearings. To verify the essentiality of preprocessing the rolling bearing vibration signals using the VMD, some contrast experiments between VMD and other traditional signal decomposition method, such as EMD, have been carried out. Here, uncertainty problems, such as measuring error, calculating error and systemic error, are considered. Vibration signals are described in the form of the generalized interval by general error ±5%. At first, the vibration signal was decomposed by the VMD and EMD methods. After signal decomposition, the MPE technique is used to calculate the main decomposed sub-signal. Using the PCA technique, major generalized PCs were obtained. At last, the GHMM model was used to recognize and classify the fault types of defect bearings. In the contrast experiments, the effect of using different numbers of major PCs was also discussed.

Results of the comparative experiments are shown in Table 6. It shows that the VMD method is much more effective than the EMD, not only in the training datasets, but also in the testing datasets. For example, using 4, 5, 6, 7 and 8 PCs, the VMD method can almost recognize and classify all of the fault types with an accuracy rate of 100%. In the EMD, choosing the main intrinsic mode function causes information loss. To some extent, the EMD technique weakens the recognition ability of the proposed method. However, the VMD technique is used to denoise the raw vibration signals and extract the frequency domain of interest.

To illustrate this point, the circumstance without signal decomposition is also investigated. The MPE is directly applied to calculate the PE values of the original generalized vibration signals. The recognition results are also shown in Table 6. The proposed method without the signal decomposition can identify the fault types of defect bearings with an accuracy rate of about 100%. It has a poorer classification ability than the VMD-based method in testing datasets. Due to containing full defect information, it performs better classification than the EMD-based method when 3, 4, 6, 7 and 8 PCs are used. Based on the comparison experiments, it can be found that the generalized vibration signal needs to be decomposed firstly. Furthermore, the ability of recognizing and classifying the fault types of the defect bearings is improved by the VMD.

#### 4.6.2. Improvement in Features Extraction

In order to verify the superiority of multi-scale analysis with the MPE, a detailed comparison is conducted using different feature extraction methods, such as PE and Shannon entropy (SE). Here, epistemic uncertainty is paid attention. The balancing parameter α is set in the form of the generalized intervals [1200, 2000] to quantify the epistemic uncertainty. At first, the vibration signal was decomposed by the VMD. After signal decomposition, different feature extraction methods are used to calculate decomposed sub-signals as follows:

- MPE-based extraction: The MPE with time scale factor 20 is used to extract features from the third decomposed sub-signal, and then, the PCA reduces the feature dimension into three PCs.
- PE-based extraction: The PE is used to calculate the third decomposed sub-signal.
- PE/SE ratio-based extraction: The PE and SE are used to calculate each decomposed sub-signal, and the entropy ratio is chosen as the features [19,43].

At last, the GHMM model was used to recognize and classify the fault types of defect bearings.

Results of the contrast experiments are shown in Table 7. The MPE-based extraction method only using three PCs is more effective than the PE-based method, not only in the training datasets, but also in the testing datasets. Due to its single scale algorithm, the PE-based method has limited performance in analyzing complicated vibration signals. The MPE-based method can extract multi-scale defect information from the decomposed sub-signal.

The entropy ratio-based method has been used to analyze the vibration signal and identify the machine states in different working conditions [18,19]. Similarly, PE/SE ratio-based extraction is used here. Based on results shown in Table 7, PE ratio-based extraction improves the ability of recognition more than the PE-based method. However, it still performs worse than the MPE-based extraction. On the other hand, SE ratio-based extraction has a similar recognition ability compared to the MPE-based method. In turn, the effectiveness of the proposed MPE-based method has been verified. Furthermore, combining the results shown in Table 5, the proposed MPE-based method using other different numbers of PCs is much better. Thus, the improvement of feature extraction using the proposed MPE-based method has been demonstrated.

#### 4.6.3. Improvement in Pattern Recognition

Similarly, these samples in Table 4 are also tested using traditional HMM. Recognition results using HMM are shown in Table 8. Based on the maximum log-likelihoods criterion [44], the recognition results are highlighted in bold. Compared to the results in Table 8, the recognition result of sample No. 3 is incorrect. It is recognized as the inner race defect condition. Thus, using the maxi-max criterion (optimistic criterion), the accuracy rate of recognition can be improved. The comparison shows that the proposed hybrid GHMM-based recognition methodology is more reliable than the HMM.

Furthermore, logp(O|λ) is a form of generalized interval probability in the GHMM. With much more information provided by the interval values, the GHMM can improve the reliability of recognition. The width of an interval probability helps to quantify the extent of epistemic uncertainty. For instance, [−11.965, −54.34] overlaps with [−9.16, −12.445] in Table 4. It provides the hidden information that the mode could be possibly misinterpreted. Thus, in order to make a robust decision, more experiments should be conducted. So much more data can be obtained to analyze and deal with this problem. In contrast, using the HMM cannot obtain such hidden information.

A comparative study between the present work and some published literature is presented to demonstrate the effectiveness and potential application of the proposed hybrid GHMM-CM method in bearing fault type identification, as shown in Table 9. The comparing items include the fault type, decomposition technique, feature extraction technique, feature selection technique, classifier and model, maximum classification efficiency and the reference.

### 4.7. Fault Severity Levels’ Classification

Recognition and classification of different fault severity levels were also performed. For the defect bearing with inner race defect and ball defect, the fault diameters are 7 mil, 14 mil, 21 mil and 28 mil; while, the fault diameters are 7 mil, 14 mil and 21 mil for the defect bearing with the outer race defect. Vibration signals are divided into non-overlapping segments with the length *N* = 2400. Similarly, each fault diameter has 50 samples, in which 25 samples will be chosen as the training dataset using the Kennard and Stone algorithm [33,34]. The remaining 25 samples are used to test the constructed system model. Here, the balancing parameter α of the VMD was set in the form of the generalized intervals [1200, 2000] to quantify the epistemic uncertainty. Eight PCs were chosen to extract generalized features from fault diameter data. Outputs of the GHMM were set to State 1, State 2, State 3 and State 4 corresponding to 7 mil, 14 mil, 21 mil and 28 mil, respectively.

**Figure 9:**
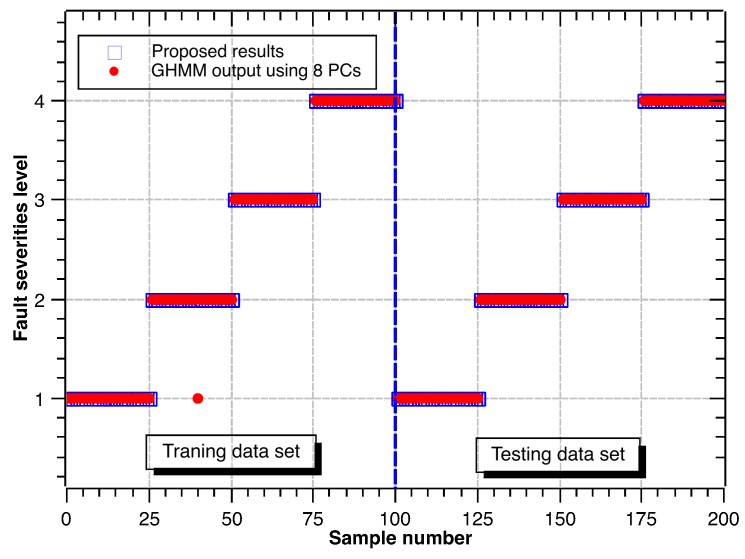
Classification results of fault severity levels under inner race defect.

**Figure 10:**
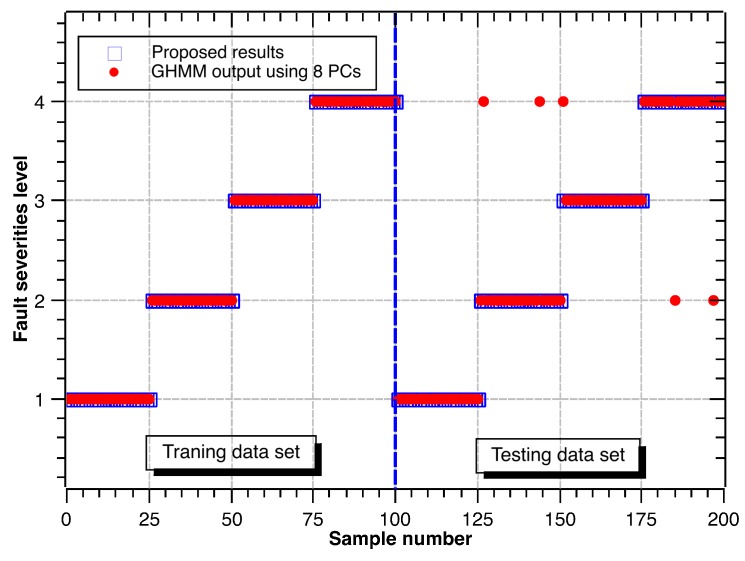
Classification results of fault severity levels under ball defect.

**Figure 11:**
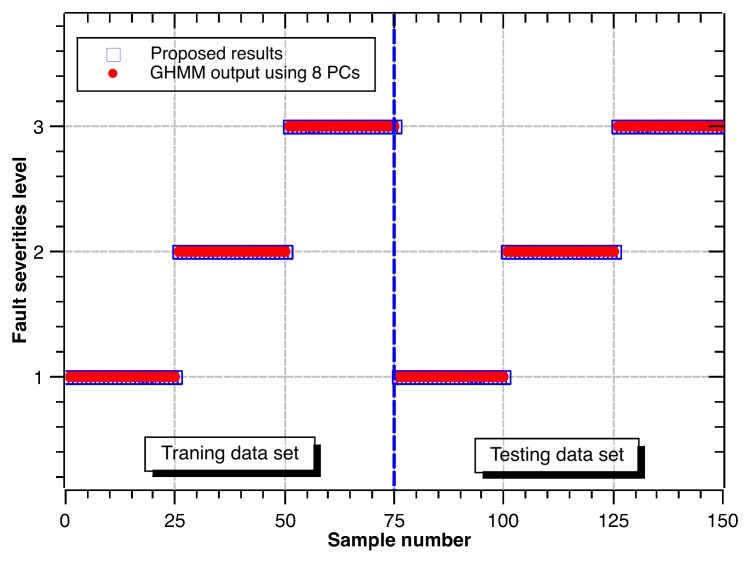
Classification results of fault severity levels under outer race defect.

Following the same procedure above, the recognition results for different fault diameters are shown in Figure 9, Figure 10 and Figure 11. Accuracy rates for different fault diameters are shown in Table 10. The ability of recognition and classification is still effective. The accuracy rates of fault severity levels are almost above 96.0% for different fault types. Thus, the proposed hybrid GHMM-CM method can also identify fault severity levels of defect rolling bearings.

## 5. Conclusions

In this paper, we proposed a hybrid GHMM-CM method using reduced decomposition features for fault types and fault severity level state recognition and classification. Vibration signal with defect information was decomposed into multiple mode components by the VMD method, in which the generalized balancing parameter provides a concise representation for aleatory and epistemic uncertainty. Then, the MPE technique extracts the interval valued features from the decomposed sub-signal. These features are closely related to defect information of rolling bearings. Next, the PCA technique was applied to reduce the dimensionality of features and computational cost. Further, identified fault types and fault severity levels of the rolling bearings based on classified features were recognized. Experimental results show that the proposed hybrid GHMM-CM method is more accurate and reliable. At the same time, this monitoring approach is efficient enough to quantify the two uncertainty components. It also provides a basic frame to deal with similar problems in other fault diagnoses and may be attractive for other application fields, such as the milling process, gearbox, wind generation, multi-agent systems, etc.

## Figures and Tables

**Table 1:** Experimental settings of machine rotation experiments.

Types	Values
Fault types	inner race defect, ball defect, outer race defect
Fault severity levels	7 mil, 14 mil, 21 mil, 28 mil
Rotation speed	1730 rpm, 1750 rpm, 1772 rpm, 1797 rpm
Sampling frequency	12 kHz

**Table 2:** Geometrical parameters of 6205-2RS JEM SKF.

Types	Values
Rolling element number (N)	9
Ball diameter (d)	312.6 mil
Pitch diameter (D)	1537 mil
Contact angle (α)	0

**Table 3:** Bearing defect frequencies under the rotating speed of 1750 rpm.

Types	Values
Shaft rotation frequency ($f_{r}$)	29.17 Hz
Inner defect frequency ($f_{id}$)	157.95 Hz
Ball defect frequency ($f_{bd}$)	137.48 Hz
Outer defect frequency ($f_{od}$)	104.56 Hz

**Table 4:** Recognition results of fault types based on the GHMM; normal condition (NC), inner race defect condition (IRDC), ball defect condition (BDC) and outer race defect condition (ORDC).

Sample No.	Optimal GHMM for NC	Optimal GHMM for IRDC	Optimal GHMM for BDC	Optimal GHMM for ORDC
1	**[−0.0842, −0.0412]**	[−Inf, −1617.4]	[−172.79, −582.69]	[−Inf, −1949.5]
2	[−Inf, −Inf]	**[−2.248, −51.994]**	[−94.4, −110.13]	[−Inf, −97.352]
3	[−Inf, −Inf]	[−Inf, −623]	**[−108.19, −4.997]**	[−Inf, −639.01]
4	[−921.2, −Inf]	[−424.76, −165.2]	[−11.965, −54.34]	**[−9.16, −12.445]**

**Table 5:** Accuracy rate using different principal components.

Number of PCs	Training	Testing	Total
8	100.0%	98.0%	99.0%
7	100.0%	98.0%	99.0%
6	100.0%	98.0%	99.0%
5	100.0%	99.0%	99.5%
4	100.0%	100.0%	100.0%
3	100.0%	94.0%	97.0%

**Table 6:** Accuracy rate considering the effect of the signal decomposition methods. EMD, empirical mode decomposition.

Signal Decomposition		3 PCs	4 PCs	5 PCs	6 PCs	7 PCs	8 PCs
	Training	100.0%	100.0%	100.0%	100.0%	100.0%	100.0%
VMD	Testing	96.0%	100.0%	100.0%	99.0%	100.0%	100.0%
	Total	98.0%	100.0%	100.0%	99.5%	100.0%	100.0%
	Training	91.0%	96.0%	97.0%	97.0%	95.0%	95.0%
EMD	Testing	89.0%	94.0%	95.0%	94.0%	92.0%	93.0%
	Total	90.0%	95.0%	96.0%	95.5%	93.5%	94.0%
	Training	100.0%	100.0%	100.0%	100.0%	99.0%	100.0%
Without signal decomposition	Testing	95.0%	93.0%	91.0%	91.0%	95.0%	98.0%
	Total	97.5%	96.5%	95.5%	95.5%	97.0%	99.0%

**Table 7:** Accuracy rate considering the effect of the feature extraction methods. MPE, multiscale permutation entropy; SE, Shannon entropy.

Feature Extractions	Training	Testing	Total
MPE-based extraction	100.0%	94.0%	97.0%
PE-based extraction	90.0%	84.0%	87.0%
PE ratio-based extraction	94.0%	93.0%	93.5%
SE ratio-based extraction	100.0%	94.0%	97.0%

**Table 8:** Recognition results of fault types based on the HMM.

Samples No.	Optimal HMM for NC	Optimal HMM for IRDC	Optimal HMM for BDC	Optimal HMM for ORDC
1	**−0.67167**	−1482.5	−649.79	−Inf
2	−Inf	**−5.7906**	−75.438	−Inf
3	−Inf	**−497.99**	−657.96	−Inf
4	−Inf	−115.62	−83.03	**−2.1655**

**Table 9:** Comparative studies for recognizing and classifying fault types of defect bearings.

Fault Type	Preprocess	Feature Technique	Feature Selection	Classifier and Model	Maximum Classification	References
ORD, IRD, BD	LMD	MSE	N/A	SVM	100.0%	Liu and Han [45]
ORD, IRD, BD	EMD	Statistics	N/A	PSO-SVM	97.5%	Liu et al. [46]
ORD, IRD, BD	N/A	MPE	N/A	SVM	100.0%	Wu et al. [26]
ORD, IRD, BD	Wavelet	Different attribute filters	N/A	SVM and ANN	97.5%	Vakharia et al. [47]
ORD, IRD, BD	LMD	MPE	LS	SVM-BT	100.0%	Li et al. [48]
ORD, IRD	LCD	Energy entropy	N/A	ACROA-SVM	100.0%	Ao et al. [49]
ORD, IRD, BD	NA	Multi-scale analysis	MD	SVM	99.79%	Wu et al. [50]
ORD, IRD	EMD	Energy entropy	N/A	ANN	93.0%	Yu et al. [51]
ORD, IRD, BD	VMD	AR	N/A	RF	100.0%	Han et al. [52]
ORD, IRD, BD	VMD	MPE	PCA	GHMM	100.0%	present work

**Table 10:** Accuracy rate of recognition for different fault diameters.

Fault Types	Fault Diameters	Training	Testing	Total
IRD	7 mil	100.0%	100.0%	100.0%
	14 mil	96.0%	100.0%	98.0%
	21 mil	100.0%	100.0%	100.0%
	28 mil	100.0%	100.0%	100.0%
BD	7 mil	100.0%	100.0%	100.0%
	14 mil	100.0%	92.0%	96.0%
	21 mil	100.0%	96.0%	98.0%
	28 mil	100.0%	92.0%	96.0%
ORD	7 mil	100.0%	100.0%	100.0%
	14 mil	100.0%	100.0%	100.0%
	21 mil	100.0%	100.0%	100.0%
